# Movement behaviour of two social urticating caterpillars in opposite hemispheres

**DOI:** 10.1186/s40462-020-0189-x

**Published:** 2020-01-31

**Authors:** Mizuki Uemura, Lynda E. Perkins, Myron P. Zalucki, Andrea Battisti

**Affiliations:** 1 Department of Agronomy, Food, Natural resources, Animals and Environment, University of Padova, 35020 Legnaro, Padova, Italy; 2 School of Biological Sciences, The University of Queensland, St Lucia, Queensland 4072 Australia

**Keywords:** Australia, Europe, Lepidoptera, Medical importance, *Ochrogaster lunifer*, Processionary caterpillars, Pupation site, *Thaumetopoea pityocampa*

## Abstract

**Background:**

Investigating movement ecology of organisms has economic, societal, and conservation benefits. Larval movement of insects for example, plays many significant ecological roles, and with the expansion of the human population and development, encounters and conflicts with insects have increased. Urticating caterpillars are a health concern to people and animals, especially when they disperse in a gregarious and synchronised manner in areas frequented by humans. *Ochrogaster lunifer* and *Thaumetopoea pityocampa* from the southern and northern hemispheres respectively, are two geographically-isolated species of moth with similar gregarious urticating caterpillars that can outbreak causing defoliation and medical issues.

**Methods:**

Each year from March to May, *O. lunifer* and *T. pityocampa* caterpillars leave their nesting sites and form head-to-tail processions on the ground in search of pupation sites. This pre-pupation procession behaviour and its associated risk of human contact with *O. lunifer* and *T. pityocampa* caterpillars were studied and compared in Australia and Italy, respectively. The distance, duration, orientation and response to visible light of the pre-pupation processions were studied in both species to determine general patterns.

**Results:**

In the morning, *O. lunifer* and *T. pityocampa* processions travelled on average 40 and 16 m per day from the nest in 153 and 223 min respectively, in search for potential pupation sites. *Ochrogaster lunifer* pre-pupation processions travelled generally to the north or south when leaving the nest, as was their final orientation to the bivouac/pupation site. Whereas *T. pityocampa* processions had no preference in orientation. *Ochrogaster lunifer* and *T. pityocampa* pre-pupation processions travelled towards the darker and the lighter areas of the environment, respectively. During our observations, 27% of *O. lunifer* and 44% of *T. pityocampa* processions had contact with humans driving, cycling or walking.

**Conclusions:**

The amount of human contact is surprising and alarming, because of the serious health implications they cause to humans and animals. The processionary dispersal on the ground risks further spread of urticating hairs that can be easily detached, and particular during inadvertent contact. Our limited sample size of *T. pityocampa* processions may benefit from more observations to make conclusive remarks on their pre-pupation behaviour*.* Understanding the movement behaviour of *O. lunifer* and *T. pityocampa* pre-pupation processions around populated areas is crucial for predicting exposure risk and application of management strategies.

## Background

The need to understand animal movement has increased over the years, whether it is for pest management, novel ecological discoveries, conservation and protection of a species, or impacts of anthropogenic factors and climate change on organisms, just to name a few. As humans modify the environment and expand its use for agriculture, housing and recreation, encountering wildlife is not out of the ordinary. Investigating movements of black bears by Zeller et al. [1] highlighted the importance of conservation and management of these species in human development. On the other hand, human-induced environmental change has exacerbated the spread of the highly invasive fire ants in United States [2]. The occurrence and spread of the red imported fire ant and its impacts on humans and endangered species native to the area were examined to protect and help recover endemic populations of organisms that were affected [2]. Further understanding of movement ecology has economic, societal, and conservation benefits.

Larvae of many Lepidoptera (butterflies and moths) leave their larval feeding sites to find suitable pupation sites and this stage is when the maximal larval movement occurs [3]. Larval movement plays many important ecological roles, and with the expansion of the human population and development, encounters and conflicts with pest insects have increased. Pest insects are defined as organisms that cause harm to humans, animals, crops or property [4]. One of which includes gregarious caterpillars associated with defensive structures such as urticating hairs which can be in the form of true setae, modified setae, or spines [5]. Urticating hairs are used as a defence against natural enemies and living in large groups has been hypothesized to enhance this mechanism [6]. True setae are a characteristic of processionary caterpillars of Notodontidae and tussock moths (Erebidae) and can also occur on the adult of a few species [7]. The setae are released by disturbance and/or mechanical stimulation and are harmful to humans and animals in several ways, including urticaria and allergic reactions in humans, tongue necrosis in dogs [8], and miscarriages in horses [9]. Occurrences of urticaria in humans from processionary moths accounts for 6 to 18% of the population in Europe [5]. In Australia, numerous cases of urticaria in humans by *O. lunifer* larvae have been reported by Southcott [10] and in literature as early as 1911 [11] (in both cases as *Teara contraria*).

Humans and animals can be exposed to urticating setae by direct contact with larvae, indirectly via the environment (e.g. on the ground and carried by wind), and by ingestion through contaminated feed and water [8]. The dispersal of urticating caterpillars during pre-pupation processions is one of the highest risk times for direct contact with humans and animals [12, 13]. In a pre-pupation procession, gregarious caterpillars ready to pupate travel head-to-tail in a line from their nest to a pupation site (Additional file 1). Caterpillars maintain a procession by following silk threads, trail pheromone and through thigmotaxis [14, 15]. The leader (first larva of the procession) and the following caterpillars are kept in contact by the long posterior hairs from the last abdominal segment and the head [15, 16]. Caterpillars aggregating in groups for at least part of their larval stage is common, however being gregarious at all instars from neonate to pre-pupae is uncommon [17]. Social species from the Notodontidae, *Ochrogaster lunifer* Herrich-Schäffer and *Thaumetopoea pityocampa* (Denis & Schiffermüller) are two examples of the latter*. Ochrogaster lunifer* and *T. pityocampa* have a univoltine lifecycle, with larvae feeding throughout the summer and winter, respectively. Every year, from March to May, pre-pupation processions of *O. lunifer* and *T. pityocampa* occur in southern and northern hemispheres, respectively. Although geographically isolated, the two species share a similar period of development through the year but in opposite seasons [18, 19].


**Additional file 1** Video MOV Time lapse of a *Thaumetopoea pityocampa* pre-pupation procession in search for a suitable bivouac/pupation site. Video filmed by Mizuki Uemura using an Apple iPhone 8.


Pre-pupation dispersal has interested entomologists but where they go has raised recurring questions [20]. Behaviour of pre-pupation processions of *O. lunifer* [18, 21, 22] and *T. pityocampa* [16, 23, 24] have been described however, all studies contained no quantitative data and analyses of the processions. *Ochrogaster lunifer* pre-pupation processions travel several days until they find a suitable pupation site [18] which can be up to 200 m from the host tree [22]. After leaving the host tree, the procession can split into smaller groups containing 1 to 10 larvae that disperse and pupate over a large area [18]. There is more literature on the behaviour of *T. pityocampa* pre-pupation processions compared to *O. lunifer*. It is known that *T. pityocampa* larvae descend from the tent in the tree canopy and reach the ground by 07:00 (solar time), with most activity on the ground between 07:00 and 09:00 [23]. There is little to no pre-pupation procession activity during cold and rainy days [23]. The number of larvae in a procession varies and splitting into smaller groups occurs for undetermined reasons but occurs more frequently when larvae are traveling on rough ground [23]. The leader of the procession is positively phototactic and will therefore lead the following caterpillars to a location where it is lighter [16], but not directly towards the sun [24]. *Thaumetopoea pityocampa* processions travel on average 16.5 m at a speed of 4.1 m/h to their pupation site across 2 days [23]. When the leader finds a suitable pupation site, all the larvae from the procession burrow together and spend one or 2 days in the top layer of the soil before they go further underground to pupate [23].

Here we investigate the behavioural movement of *O. lunifer* and *T. pityocampa* pre-pupation processions and the occurrences of human contact. The study focused on quantitative observational and environmental data associated with pre-pupation procession movements. It is important to understand if the two model species disperse in a predictable way and how general this behavioural pattern may be in the same taxonomical group. Knowledge of dispersal movements in these important pest species will facilitate building models to predict exposure risk and application of pest management strategies.

## Methods

### Field sites and data collection

#### Ochrogaster lunifer

*Ochrogaster lunifer* is a widespread univoltine species found across coastal and inland Australia where its *Acacia*, *Eucalyptus* and *Corymbia* spp. host trees occur [12, 25]. Within the species, there are five nesting types [12] with different ecology, morphology and genetics [26]. In our study, we focused only on the ground nesting form in which larvae create a silken nest at the trunk base of various *Acacia* and *Eucalyptus* spp. host trees [18]. The larvae feed throughout the summer until they reach their final instar in autumn, when they leave their nest permanently in search of a suitable pupation site underground. In our study, *O. lunifer* pre-pupation processions were followed in two seasons, one over seven and the other over ten non-consecutive days from end of March until first week of April 2017 and 2019, respectively. Field visits occurred on other days during the season but there were no active processions. Processions came from various *Acacia* spp. tree ground nests at The University of Queensland (UQ), Gatton campus, Queensland, Australia (− 27°56′S, 152°34′E, Additional file 2). The campus is a semi-urban environment with buildings and patches of vegetation throughout. Larvae generally left the nest after sunrise, between 06:00 to 07:00 local time. Time at which the leader of the procession left the nest and the time at which the last larva of the procession (or singleton) went underground (bivouac) were recorded (Additional file 3). A bivouac is not necessarily the final site where the larvae pupate, especially for the first day of travel (M. Uemura, personal observation 2017). Most often, the larvae leave the bivouac at the following sunrise to a new bivouac/pupation site. Any disruptions during the procession were recorded, e.g. larvae run over by cars or pedestrians and were classified as human contact. Every time a procession broke into sub-processions or changed direction, coloured flags with codes were used as markers. Once the procession and/or singleton went underground, the GPS coordinates of flags and where caterpillars went underground were recorded with Garmin GPS Etrex10 (in 2017) or iPhone application ViewRanger (in 2019). In-situ environmental temperatures were recorded for *O. lunifer* populations using Tinytag Plus 2 data-logger TGP-4505 (Hastings Data Loggers, Port Macquarie, Australia) in 2017 but not in 2019. Environmental temperatures for 2019 during the pre-pupation processions were collected from the UQ Gatton station 040082 [27].


**Additional file 3** Video MOV Time lapse of a *Thaumatopoea pityocampa* pre-pupation procession going underground into a bivouac. Video filmed by Mizuki Uemura using an Apple iPhone 8.


#### Thaumetopoea pityocampa

*Thaumetopoea pityocampa* larvae are destructive defoliators of pine and cedar trees in the Mediterranean Basin and Southern Europe [28]. *Thaumetopoea pityocampa* larvae feed throughout winter until spring in northern Italy, when the final instar larvae leave their tent (nest) in the canopy and descend to the ground to find a pupation site [23]. *Thaumetopoea pityocampa* pre-pupation processions were followed over seven and two non-consecutive days from the first until the third week of April 2018 and last week of March until first week of April 2019, respectively. Field visits occurred on other days during the season but there were no active processions. The field sites were at three pine forests in Veneto, Italy: Monte Garzon (45°30′ N, 11°11′ E, Additional file 4), Precastio (45°31′ N, 11°10′ E) and Carbonari (45°32′ N, 11°10′ E). The selected field sites are planted forest for soil conservation and are also used for recreational purposes such as hiking, cycling and picnics. Processions could not be followed from the tent because they are situated in the canopy of *Pinus nigra* host trees that can be more than 6 m high. Therefore, procession data could only be collected from the first sighting along the footpath of the field sites, i.e. all *T. pityocampa* pre-pupation procession data are approximate measures. Once a procession was found and followed until the bivouac, GPS of the bivouac location was recorded using iPhone application ViewRanger and any disruption/movement of the procession were recorded in a similar manner to the *O. lunifer* processions. Continuous environmental temperatures for *T. pityocampa* field sites were measured from HOBO Temperature/RH data loggers (Onset Computer Corporation, Macquarie, USA) placed in a village approximately 1 km away from each field site (Tregnago, Veneto, Italy).

### Distance, duration and speed of pre-pupation processions

Topographic distance and travel duration by *O. lunifer* were calculated from processions leaving the nest until completing a bivouac. Once all the larvae from a nest were underground, the distance travelled was measured with a measuring tape or trundle wheel, retracing every movement of the procession. Speed was calculated from the duration and distance travelled from the nest or first sighting to the bivouac. Distance and duration travelled by *T. pityocampa* pre-pupation processions are an underestimate because it was not feasible to follow processions from *T. pityocampa* tents that are in the tree canopy. The distance travelled by *T. pityocampa* was calculated by addition of the height of the nearest host tree with a visible viable nest (estimated from the orientation at first sighting of the procession before it formed a bivouac) and distance from that host tree until the bivouac site. Duration of travel was calculated by dividing the estimated distance travelled by the average speed of *T. pityocampa* pre-pupation processions. Speed was calculated from measurements of 15 processions on the ground (e.g. travelled x m in x mins).

### Orientation of pre-pupation processions

Orientation of every procession is determined by the leader. Each directional change of the procession leaving from the nest to the bivouac was recorded using a handheld compass (in Australia 2017) or iPhone application Compass (in Italy 2018/19 and Australia 2019). For *O. lunifer*, orientation of all pre-pupation processions leaving the nest and final orientation to the bivouac following the last turn of the leader were used for the data analyses. For *T. pityocampa*, orientation of processions at first sighting were used for the data analyses.

### Light preference of leading larvae

Solar radiation/light (W/m^2^) was measured for a sub-sample of 2018 *T. pityocampa* and 2019 *O. lunifer* processions using the Solar Power Meter (SPM) ISM410 (RS Pro, 2016, London, England). Solar radiation was measured for *O. lunifer* processions when they left the nest and at final orientation to the bivouac. For *T. pityocampa* processions, the solar radiation was measured at first sighting. Three solar radiation measurements were taken: directly beside at the same orientation as the leader, 90^o^ left and right of the leader. Standardised light was calculated by dividing light (W/m^2^) from the leader’s position by the average of left and right of the leader. Value of 1 means there is no difference between the light intensity at the orientation of the leader/procession and the surrounding. Values more or less than 1 means the leader/procession travelled to the lighter or darker relative to the surrounding environment, respectively.

### Risks of pre-pupation processions contacting humans

For *O. lunifer*, 2019 data for caterpillar contacts with humans were used, because in 2017, some processions were protected from being run over by cars and walked over by pedestrians. Processions for *T. pityocampa* were not protected from encounters by humans. A human attended area is defined as areas where people travel to and from places by walking, cycling and driving. It was calculated so the two field sites, Australia and Italy can be compared. For *O. lunifer*, the average human attended area in percent at UQ Gatton campus was calculated by the amount of urban area (e.g. concrete footpath, buildings, roads, etc.) surrounding each host tree at a 10 m radius using QGIS version 3.6.2 Noosa [29] (10 host trees in total). Human attended areas at UQ are not suitable for bivouac/pupation sites because it is made of concrete; with the exception of some areas that had leaf litter. For *T. pityocampa*, the average human attended area in percent was calculated at Monte Garzon by the amount of gravel foot path surrounding each procession at first sighting within a 10 m radius using QGIS (24 processions in total). Human attended areas in Italian field sites are the preferred and suitable bivouac/pupation sites for *T. pityocampa* because there is exposed dirt/soil and loose gravel (A Battisti, personal communication 2018).

### Statistical analyses

All statistical analyses were performed using R Studio version 1.1.419 [30] and an alpha value of *P* <  0.05 was taken as statistically significant. Mapping was performed using QGIS with satellite images from Google Earth Pro version 7.3.2 [31]. Data for *O. lunifer* populations collected in 2017 and 2019 were combined in the analyses. Linear models were used to determine if the number of larvae in a procession and environmental temperatures influenced the distance travelled and/or speed of *O. lunifer* and *T. pityocampa* pre-pupation processions. To determine if human attended areas affected the distance travelled by *O. lunifer* processions, a linear model was used, with the variables: distance travelled and human attended area of the host tree where the procession originated (Results in *Distance, duration and speed of pre-pupation processions*). Distance travelled by *T. pityocampa* processions were not modelled against human attended areas because the habitat is a pine forest. Procession orientations were represented as rose diagrams made in R Studio using the software package “Circular” [32]. To determine if processions had a preference(s) in orientation, Kuiper’s test of uniformity was used for each rose diagram with the R software package “CircStats” [33]. Each *O. lunifer* procession was nested within its host tree (10 host trees in total from 2017 and 2019 combined) therefore, Kuiper’s test of uniformity was also used to determine if host trees affected the orientation of processions. Host trees that had more than 10 processions were selected for the Kuiper’s test of uniformity. Light preference of *O. lunifer* and *T. pityocampa* pre-pupation processions were analysed using Chi-square Goodness-of-Fit test for a 50:50 distribution. A linear model was used to determine if host tree and number of larvae in the procession affected light preference by *O. lunifer* and *T. pityocampa* processions. Host tree could not be tested for light preference in *T. pityocampa* pre-pupation processions because the exact host trees and timing at which they were at the tree base were unknown.

## Results

### Distance, duration and speed of pre-pupation processions

*Ochrogaster lunifer* processions travel approximately three times further and four times faster than *T. pityocampa* (Table 1). *Ochrogaster lunifer* processions left the ground nest between 05:43–09:25 h (median = 06:15 h, *N* = 115, Additional file 5) and finished the bivouac between 06:23–14:00 h (median = 08:53 h, *N* = 86). *Thaumetopoea pityocampa* processions were found along the footpath between 07:45–14:29 h (median = 10:12 h, *N* = 38, Additional file 5) and finished the bivouac between 08:22–15:37 h (median = 13:23 h, *N* = 11). *Ochrogaster lunifer* processions travelled significantly further from the nest to the bivouac when there was more human attended area (concrete/building) surrounding the nest (LM: *t*_*85*_ = 2.810, adjusted *R*^2^ = 0.07505, *P* = 0.00616). In both species, number of larvae in a procession did not affect the total distance travelled or the speed (all *P* >  0.1). Environmental temperature at the field sites had no influence on procession speed for *O. lunifer* and *T. pityocampa* (*P* >  0.05 and *P* > 0.2, respectively) and distance travelled by *T. pityocampa* (*P* > 0.8). Environmental temperature in Gatton influenced the distance travelled by *O. lunifer*, with processions travelling less at higher temperatures (LM: *t*_*85*_ = − 3.551, adjusted *R*^2^ = 0.119, *P* = 0.000628).

**Table 1:** Comparison of the average distance travelled, duration, speed and environmental temperature between *Ochrogaster lunifer* and *Thaumetopoea pityocampa* pre-pupation processions

Species	Average distance travelled from nest to bivouac (m)	Average duration travelled from nest to bivouac (min)	Average speed of procession (m/h)	Average environmental temperature (°C)
*Ochrogaster lunifer*	40.3 (±SE 3.4, *N* = 87)	153 (±SE 11, *N* = 86)	17.4 (±SE 0.8, *N* = 129)	19.1 (±SE 0.4, *N* = 87)
*Thaumetopoea pityocampa*	15.8 ^a^ (±SE 2, *N* = 11)	223 ^a^ (±SE 30, *N* = 11)	4.2 (±SE 0.7, *N* = 15)	13.7 (±SE 0.9, *N* = 11)

^a^ Approximate measurements

### Orientation of pre-pupation processions

**Fig. 1:**
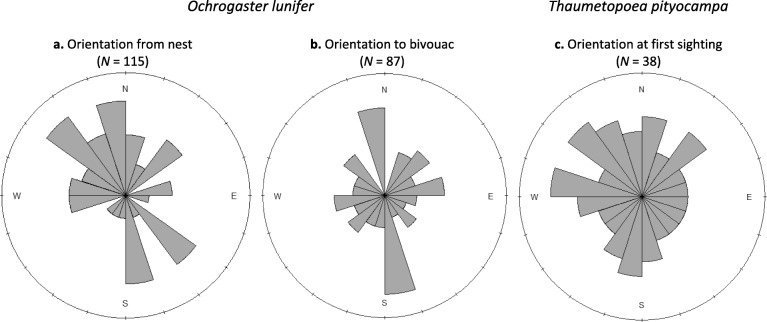
Rose diagrams of the orientation of pre-pupation processions of *O. lunifer* leaving the nest (**a**) and final orientation to the bivouac following the last turn of the leader (**b**), and of *T. pityocampa* at first sighting (**c**)

*Ochrogaster lunifer* pre-pupation processions travelled more often towards the north and south, and less to the east and west when leaving the nest and to the final orientation to the bivouac following the last turn of the leader (Fig. 1a and b, respectively; Table 2). There were six and four host trees with more than 10 *O. lunifer* processions, for orientations from the nest and to the final orientation to the bivouac, respectively. These host trees were tested for uniformity, and the tests showed that eight of the ten host trees had processions with a preferred orientation (all north and/or south, except one host tree with processions that headed to the north and west) i.e. procession orientations were not uniform and therefore clumped (all *P* <  0.025). In contrast, *T. pityocampa* pre-pupation processions had no preferred orientation at first sighting (Fig. 1c, Table 2).

**Table 2:** Comparison of Kuiper’s test of uniformity for the orientation of *O. lunifer* and *T. pityocampa* pre-pupation processions

Species	Kuiper’s test statistic	*P*	*N*
*Ochrogaster lunifer*			
Orientation from nest	2.9109	< 0.01	115
Orientation to bivouac	1.7765	< 0.05	87
*Thaumetopoea pityocampa*			
Orientation at first sighting	1.2345	> 0.15	38

### Light preference of leading larvae

**Fig. 2:**
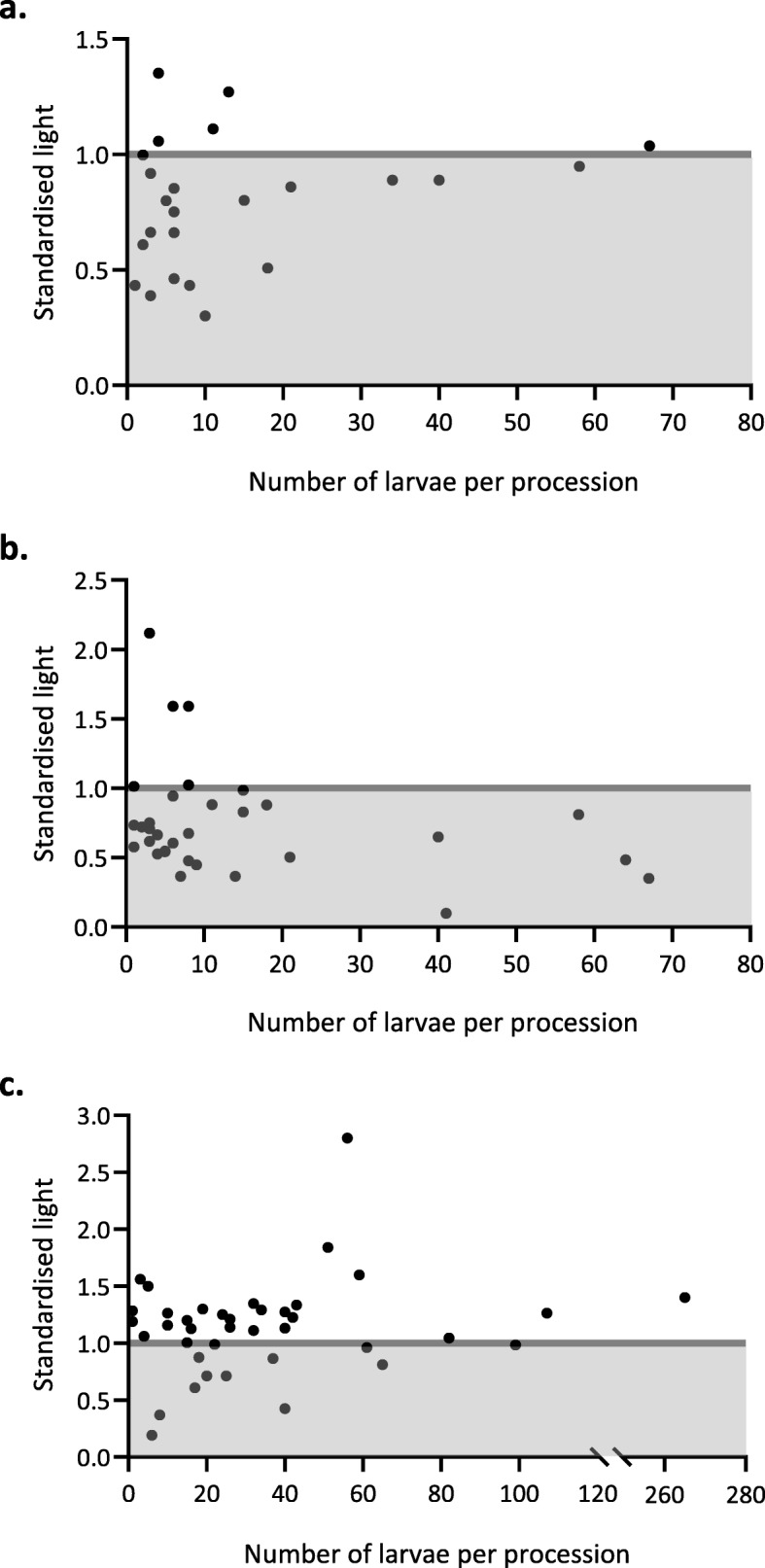
**a**. Standardised light choice by *Ochrogaster lunifer* pre-pupation processions (*N* = 24) at initial orientation when leaving the ground nest. **b**. Standardised light choice by *O. lunifer* pre-pupation processions (*N* = 33) at final orientation to the bivouac following the last turn of the leader. **c**. Standardised light choice by *Thaumetopoea pityocampa* pre-pupation processions (*N* = 38) at first sighting. Each point represents one procession. Area shaded in grey (standardised light value less than 1) represents processions that travelled to the darker in comparison to its surroundings. Unshaded area (standardised light value above 1) represents processions that travelled to the lighter compared to its surroundings. There is no correlation between number of larvae in each procession and the choice for standardised light

More *O. lunifer* pre-pupation processions travelled to the dark than to the light when leaving from the nest (*X*^2^ = 8.1667, *df* = 1, *P* = 0.004267, *N* = 24; Fig. 2a) and when arriving to the bivouac (*X*^*2*^ = 16.03, *df* = 1, *P* = 6.234e^− 05^, *N* = 33; Fig. 2b), irrespective of the number of larvae in a procession (from nest: *P* > 0.2; to bivouac: *P* > 0.1) and host tree (*P* > 0.5). Whereas *T. pityocampa* pre-pupation processions travelled more to the light than the dark (*X*^*2*^ = 5.7692, *df* = 1, *P* = 0.01631, *N* = 38; Fig. 2c), irrespective of the number of larvae in a procession (*P* > 0.4).

### Risks of pre-pupation processions contacting humans

People were 1.5 times more likely to be in contact with *T. pityocampa* than *O. lunifer* pre-pupation processions in our field sites (Table 3). On average, human attended area at UQ Gatton campus was two times larger than pine forests in Veneto, Italy. Visual representation of the procession route taken by selected *O. lunifer* and *T. pityocampa* processions from a host tree/first sighting are presented in Additional files 6 and 7, respectively.

**Table 3:** Comparison of the number of human contacts with *Ochrogaster lunifer* and *Thaumetopoea pityocampa* pre-pupation processions

Species	Number of processions with human contact	Total number of processions	Human contact (%)	Human attended area (%)
*Ochrogaster lunifer*	22	82	26.8	33 (±SE 2.0)
*Thaumetopoea pityocampa*	23	53	44	15 (±SE 0.7)

## Discussion

The expansion of human growth and development has increased the likelihood of insect-pest human interactions. This includes processionary caterpillar species *O. lunifer* and *T. pityocampa*, which can cause serious health problems to humans and animals in Australia and Europe, respectively. In our study, we focused and compared the movements of *O. lunifer* and *T. pityocampa* during the pre-pupation period to determine the risks associated to humans.

*Ochrogaster lunifer* and *T. pityocampa* pre-pupation processions travelled tens of metres per day in search for pupation sites over two or more days [18, 23]. *Ochrogaster lunifer* processions travelled 2.5 times further and 4 times faster than *T. pityocampa*. These differences could be explained by the final instar body size; *O. lunifer*: 35–50 mm [18] vs. *T. pityocampa*: 33 mm [6]; environmental temperatures (Table 1, see [34]), number of instar stages (*O. lunifer*: 8 instars vs. *T. pityocampa*: 5 instars), environmental terrain (*O. lunifer*: mostly flat and smooth grounds vs. *T. pityocampa*: loose gravel ground) and possibly other physiological and morphological differences. At the UQ Gatton campus, *O. lunifer* larvae travelling several tens of metres per day could enter paddocks containing farm animals and ingress further into human settlement; which means an increased risk for spreading setae and exposure to people and animals.

*Ochrogaster lunifer* pre-pupation processions left the nest at sunrise and travelled for approximately 2.5 h. On working days and possibly weekends, students and staff were in contact with these larvae in the morning, especially because some nests are in close vicinity to classrooms, footpaths, car parks, etc. This corresponded to the 27% of human contact with *O. lunifer* processions. In contrast, 44% of *T. pityocampa* pre-pupation processions in Italy had human contact. The chance of human contact is higher in *T. pityocampa* compared to *O. lunifer*; possibly due to the number of people that attended the area, but most likely can be explained by the behaviour of *T. pityocampa*. Human attended area in Italian field sites are approximately a half of the human attended area in Australia, and people are limited to walk only on footpaths. Additionally, *T. pityocampa* larvae do not travel far, are slow walkers and prefer lighter areas (see below) which is almost always the footpath. The longer the larvae stay on the footpath, the higher chance of contact with humans and pets. The amount of human contact in both field sites are concerning because setae are released upon disturbance [8] and the surviving larvae in the procession after contact may be very reactive. Not only do setae become airborne after release [13], it can contaminate bicycle wheels, shoes and clothing and be brought back to the car, home, classroom, etc. The toxins inside the seta can remain active for at least a year [5] and the microscopic scale of the seta makes it difficult to determine what/where is contaminated.

In general, there was a tendency for *O. lunifer* pre-pupation processions to travel north or south when leaving the nest and also to their final orientation to the bivouac. In contrast *T. pityocampa* pre-pupation processions had no preference in orientation at first sighting. The orientation preference/absence could be explained by the light preference by the leading larvae of both species. *Ochrogaster lunifer* larvae orientated towards darker areas while *T. pityocampa* orientated towards lighter areas of the environment for potential pupation sites. At the Australian field site, it is generally warmer at this time of year and host trees were patchy with a lot of clearings. Therefore, orienting more to the north or the south could avoid direct sunlight which came from the east to the west. Italian field sites were cooler because of the homogenous dense pine forest, and lighter areas are restricted to footpaths and nearby meadows. Orientation may be insignificant for *T. pityocampa* because the orientation chosen may only depend on where is lighter in relation to where the host tree is. *Thaumetopoea pityocampa* processions search for sunny exposed soil in gaps and edges of forests for optimal pupae survival [35]. Therefore, *O. lunifer* and *T. pityocampa* pre-pupation processions may have been in search for ‘cooler’ and ‘warmer’ places, respectively for optimal development in suitable thermal niches. It may be particularly important for *T. pityocampa* to find warmer places because the pupae can diapause up to 8 years [36] with possibly less chance of fungal attack if the pupation site is dryer. More research is necessary to determine if light is used by the lead larva as a cue for pupation sites with suitable temperature characteristics.

*Ochrogaster lunifer* and *T. pityocampa* processions preferred different environmental cues to navigate to the bivouac, and this may be a result of urbanisation in the environment. Organisms living in urban environments often differ in behaviour compared to those from rural environments, and having adjusted to anthropogenic disturbances [37]. This includes changes in food resources, nesting site, pedestrian and vehicle traffic, artificial light and industrial noise [37]. As environments/microhabitats for *O. lunifer* and *T. pityocampa* vary within the species, the data we present may only be representative for the field sites chosen (semi-urban and pine forest/rural, respectively). It would be beneficial to repeat the same behavioural analyses on *O. lunifer* populations in a rural/natural environment and also for *T. pityocampa* populations in a semi-urban/urban environment. This can determine whether the pre-pupation movement by the two species has indeed changed with urbanisation. *Thaumetopoea pityocampa* also has a population of summer feeding larvae in Portugal, that have pre-pupation processions occuring at the end of summer every year [38]. A comparison of winter and summer feeding *T. pityocampa* population processions could provide further information on whether or not the same behaviour and environmental preferences exists within the species.

Our results on pre-pupation processions are comparable with the findings from previous published studies. Mills [21, 39] observed *O. lunifer* processions leaving the nest in the morning in a northerly or southerly direction, consistent with our observations. Average speed of our *O. lunifer* processions (17.4 m/h at 19 °C) in the field differed from the procession work by Steinbauer [15] (approx. 23.3 m/h at 19 °C, calculated from regression of speed over temperature ‘all processions combined’). This is possibly explained by the physical handling of study organisms (posterior or anterior hairs of the larva cut or brushed) by the experimenters and/or the short observation duration of 1 min per procession (see [15] for more information). In contrast to *O. lunifer*, *T. pityocampa* pre-pupation processions travelled to lighter areas consistent with Démolin [16]; he stated that *T. pityocampa* larvae are phototactic and pupated in the open. Additionally, the distance travelled and speed of *T. pityocampa* pre-pupation processions documented by Robredo [23] were similar to our measurements (cf. Background and Table 1). However, the timing of *T. pityocampa* pre-pupation processions on the ground in our study was different to that reported by Robredo [23]. This could be explained by several cloudy and rainy days during our study which delayed the timing of caterpillars leaving the tent. Because *T. pityocampa* tents were high in their host trees, we were not able to observe caterpillars leaving their tents. Therefore, we had to use measurements from processions when they were first sighted on the ground sometime later. More observations and future investigations of *T. pityocampa* pre-pupation processions at sites with shorter host trees where the tents could be observed directly would give more accurate data on their behaviour.

The risk of exposure to *O. lunifer* and *T. pityocampa* processions are particularly high because of the tent/nest distribution in the environment. Both species are edge species, thus female moths restrict their oviposition mostly to the outer edges of forests or road verges where larval colonies later develop [35, 40, 41]. Clumped distribution of urticating caterpillars in close vicinity to human settlement, in combination with their procession movements on the ground make these species an important medical concern. Seasonal abundance or outbreaks of urticating caterpillars are recorded in other social Lepidoptera genera such as *Euproctis* (Erebidae), and *Hylesia* and *Hemileuca* (Saturniidae) [42]. These species may not form processions or travel *en masse* but they can disperse over the ground after defoliating a host plant or to find a pupation site. Precautions are needed to reduce cutaneous or systemic symptoms that can be caused by medically important Lepidoptera species (see below). Alongside the studies on black bears [1] and fire ants [2] (see Background), determining movements of organisms is crucial when it comes to human and animal interactions.

Much of the information for *O. lunifer* and *T. pityocampa* pre-pupation processions presented here is novel. Understanding the movement of urticating caterpillars in human settlement plays an important role in interpreting its ecological and medical significance. With this information, we can forewarn people to avoid infested areas especially in the morning or stay at least 80 m and 30 m away from host trees with *O. lunifer* and *T. pityocampa* nests respectively, during the pre-pupation procession period. If it is necessary to go to these high-risk areas, people should be visually alert where they are walking or cycling and wear appropriate protective clothing and eyewear. Awareness is important for prevention, because these urticating caterpillars will disperse from the host tree to pupation sites every year. With more sampling and monitoring of infested host trees, it can further improve the predictability of the movements of urticating processionary caterpillars.

## Conclusions

Anthropogenic changes in the environment are increasing and expanding, therefore more people are at risk of coinciding with insects of medical importance. We investigated and compared pre-pupation procession behaviours of two urticating caterpillar species from opposite hemispheres. Our aim was to understand their movement to determine the contact risk with humans. Differences in movement behaviour of *O. lunifer* and *T. pityocampa* pre-pupation processions may be explained by their own environmental and physiological requirements for optimal development. Our data on human contact with both species, in addition to the alarming numbers of urticaria cases in Europe, raises the need for preventative measures. The research presented here highlights the importance of investigating movement patterns of organisms to mitigate harmful impacts.

## Supplementary information

**Additional file 2 **Image PDF The University of Queensland, Gatton campus, Australia, the fieldsite where *Ochrogaster lunifer* pre-pupation processions were studied. Pre-pupation processions were followed from ten *Acacia* spp. host trees that are represented as different coloured hexagons. Processions were followed until the larvae went into a bivouac which are represented as circles (colour coordinated with the host tree). Human attended areas are shaded in white and are not suitable for bivouac/pupation sites because it is made of concrete; with the exception of some areas that had leaf litter.

**Additional file 4 **Image PDF Monte Garzon, Veneto, Italy, one of the three fieldsite where *Thaumetopoea pityocampa* pre-pupation processions were studied. Quantitative data were collected from processions at first sighting along the footpath, represented as red circles. Clearings and human attended areas shaded in white, are suitable and preferred bivouac/pupation sites for *T. pityocampa* larvae.

**Additional file 5 **Graph PDF Comparison of the observed pre-pupation procession times of *Ochrogaster lunifer* and *Thaumetopoea pityocampa* in Australia and Italy, respectively. Time of observation for *O. lunifer* (black line) was from the time the procession left the nest and *T. pityocampa* (grey line) was first sighting on the footpath.

**Additional file 6 **Images PDF A sub-sample of three *Ochrogaster lunifer* pre-pupation procession routes from one host tree at the University of Queensland, Gatton campus, Australia. Every procession starts from the host tree (yellow circle) and each arrow represents a change in procession orientation in search for a pupation site (grey/white arrows)/had human contact (red arrows). The tip of the last arrow is the last point. Red arrows represent the procession that was run over by a car on the road (human contact). Grey (A and B)/white (C) arrows represent processions that successfully went into a bivouac. In B and C, various shades of grey circles starting from the host tree is the amount of human attended areas (urban structures) there are for a given radius of various increments (5, 10, 20, 40 m). Additional file 6 A, B and C represents the same three *O. lunifer* pre-pupation processions with different geographic layers, starting from A being the simplest to C being the most complex with the satellite image.

**Additional file 7 **Images PDF. A *Thaumetopoea pityocampa* pre-pupation procession route from first sighting along the footpath at Monte Garzon, Veneto Italy. The procession was studied from the first sighting (yellow circle) and every grey (A)/white (B) arrow represents a change in orientation in search for a pupation site. The thick grey line represents the human attended area of a 10 m radius from the first sighting of the procession. Additional file 7 A and B represents the same *T. pityocampa* pre-pupation procession with different geographic layers, A being the simplest and B being the most complex with the satellite image.

## Data Availability

The data are available upon request from the authors.

## Abbreviations

UQ: The University of Queensland
