# Supplementary figures and images for: Movement behaviour of two social urticating caterpillars in opposite hemispheres
Source: Mov Ecol. 2020 Jan 31;8:4. doi: 10.1186/s40462-020-0189-x (PMC6995145; doi:10.1186/s40462-020-0189-x)

# The University of Queensland Gatton Campus

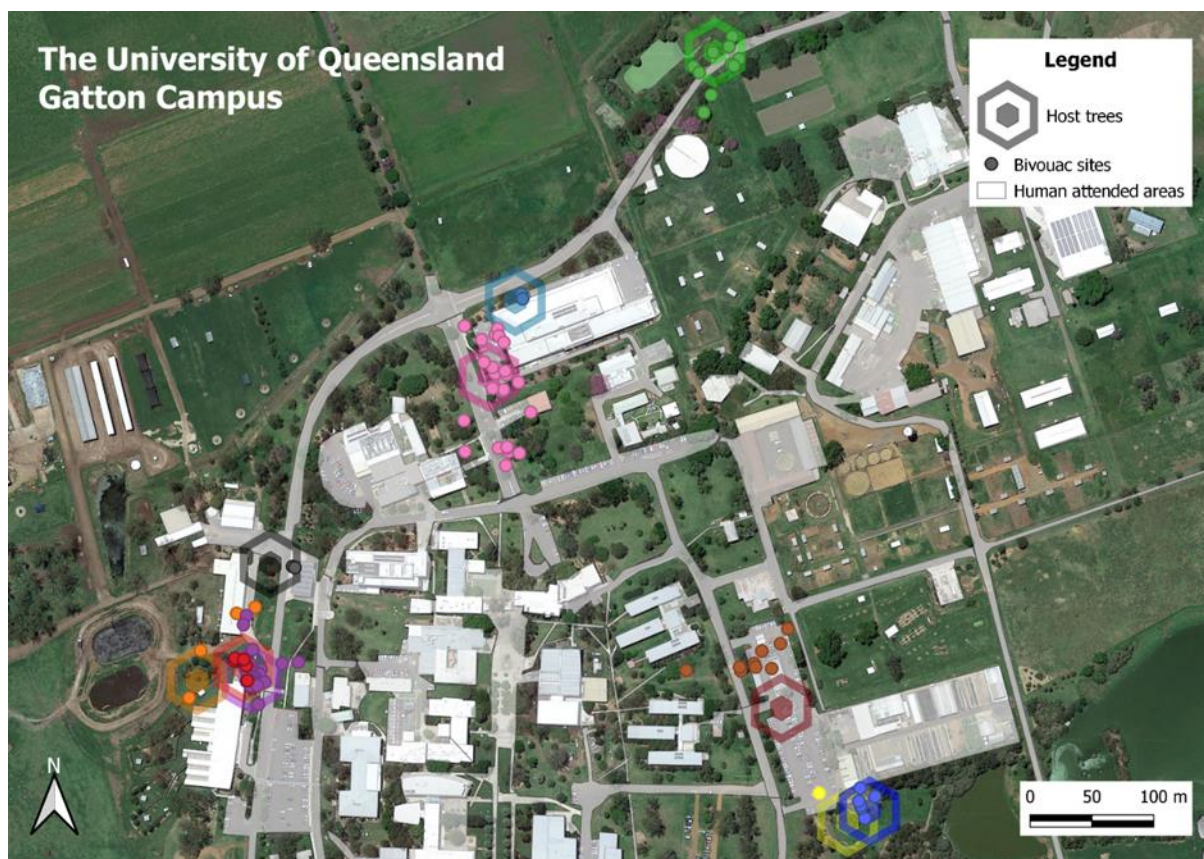

Supplement: Supplementary file 3 — Additional file 2 Image PDF The University of Queensland, Gatton campus, Australia, the fieldsite where Ochrogaster lunifer pre-pupation processions were studied. Pre-pupation processions were followed from ten Acacia spp. host trees that are represented as different coloured hexagons. Processions were followed until the larvae went into a bivouac which are represented as circles (colour coordinated with the host tree). Human attended areas are shaded in white and are not suitable for bivouac/pupation sites because it is made of concrete; with the exception of some areas that had leaf litter. [file 40462_2020_189_MOESM2_ESM.pdf]

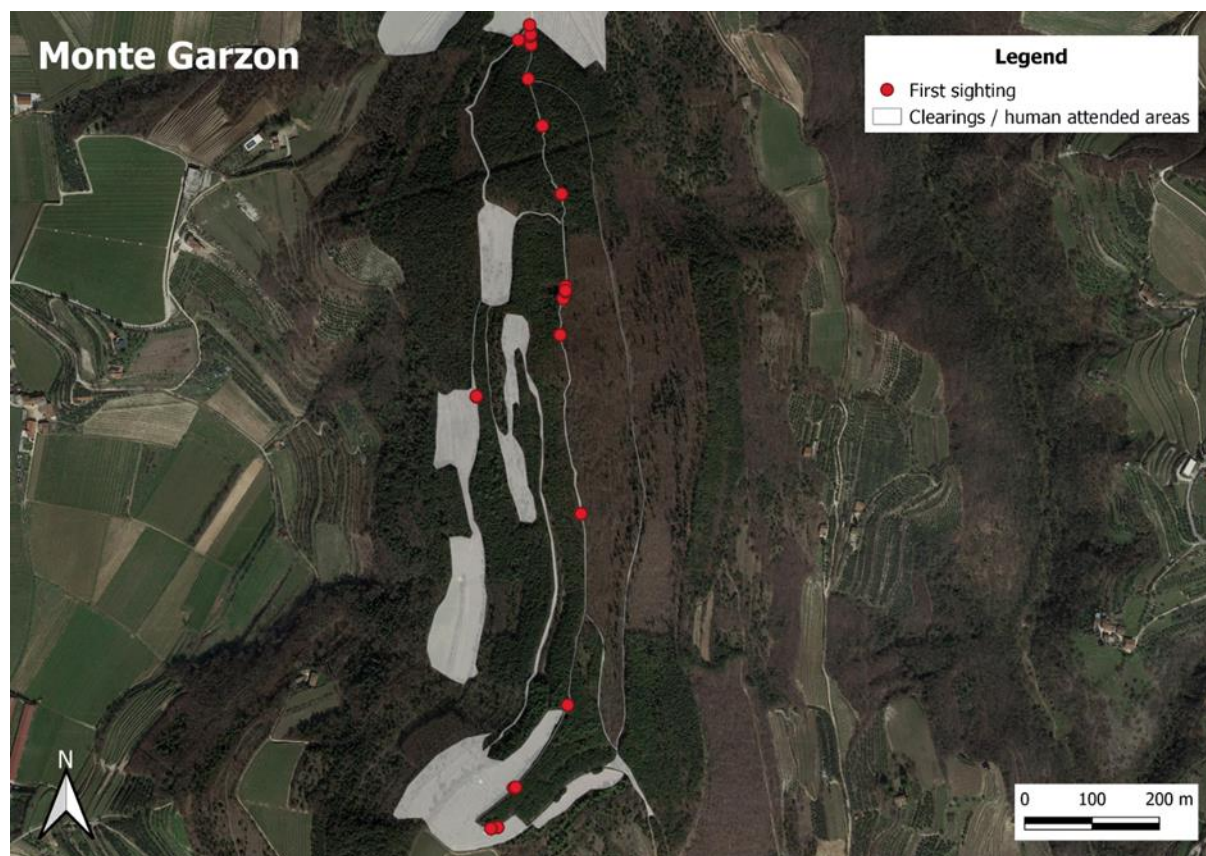

Supplement: Supplementary file 4 — Additional file 4 Image PDF Monte Garzon, Veneto, Italy, one of the three fieldsite where Thaumetopoea pityocampa pre-pupation processions were studied. Quantitative data were collected from processions at first sighting along the footpath, represented as red circles. Clearings and human attended areas shaded in white, are suitable and preferred bivouac/pupation sites for T. pityocampa larvae. [file 40462_2020_189_MOESM4_ESM.pdf]

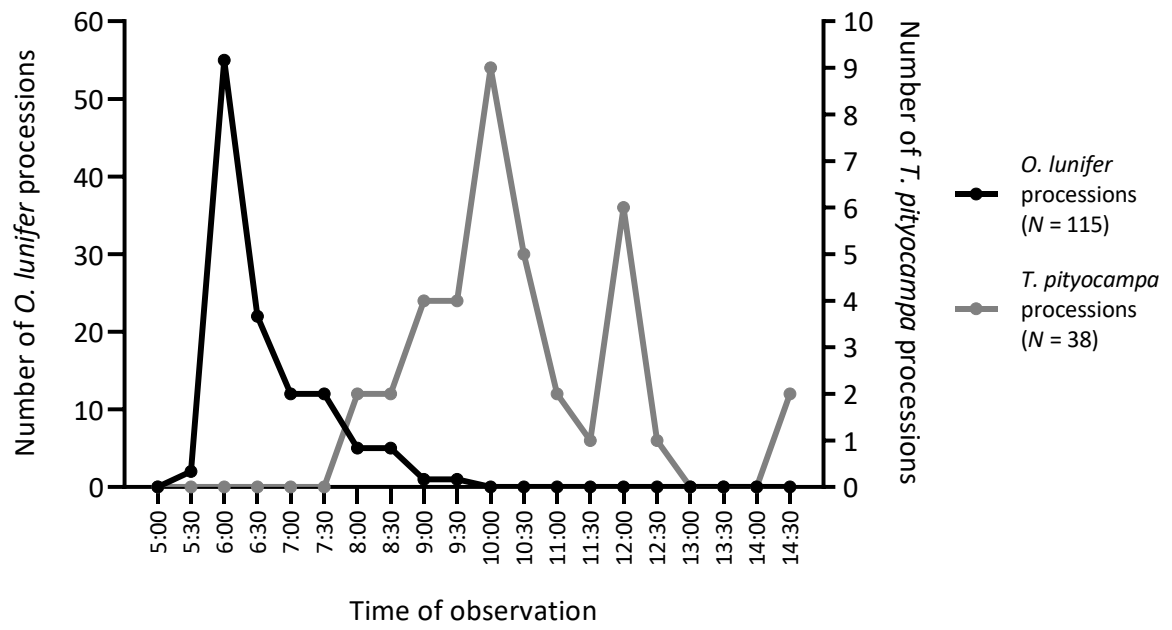

Supplement: Supplementary file 5 — Additional file 5 Graph PDF Comparison of the observed pre-pupation procession times of Ochrogaster lunifer and Thaumetopoea pityocampa in Australia and Italy, respectively. Time of observation for O. lunifer (black line) was from the time the procession left the nest and T. pityocampa (grey line) was first sighting on the footpath. [file 40462_2020_189_MOESM5_ESM.pdf]

A

## UQ Gatton Campus

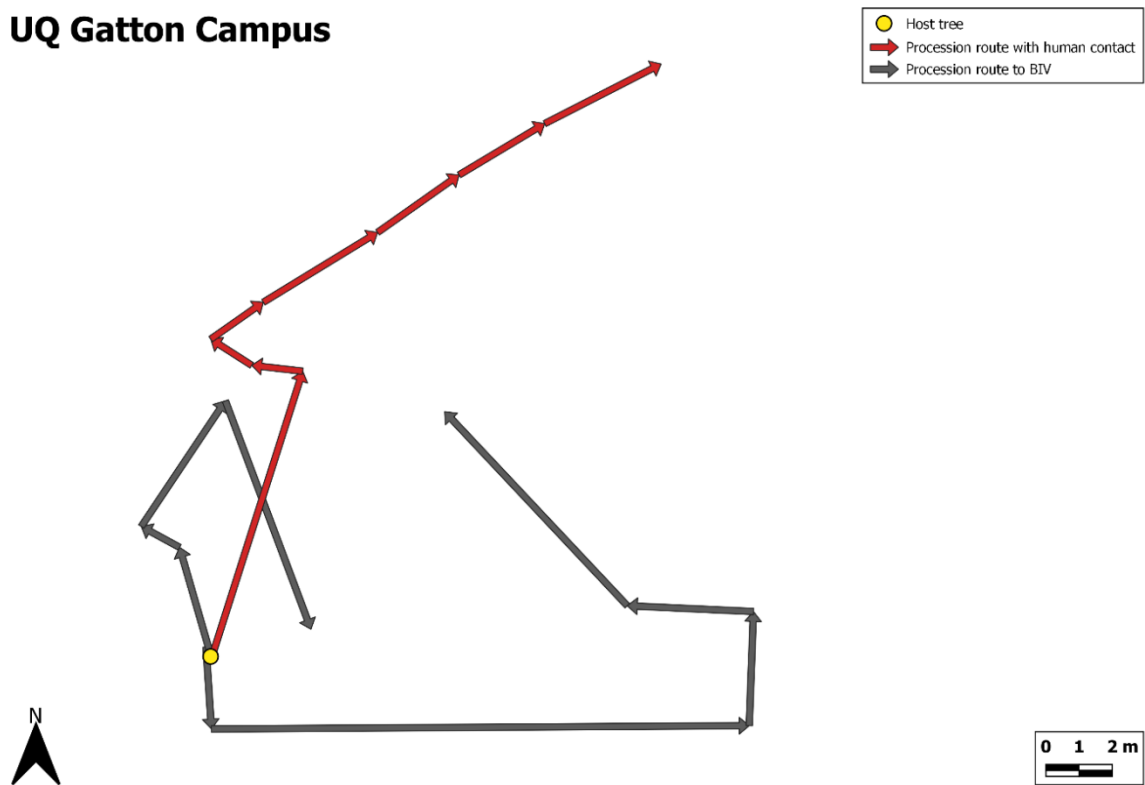

B

## UQ Gatton Campus

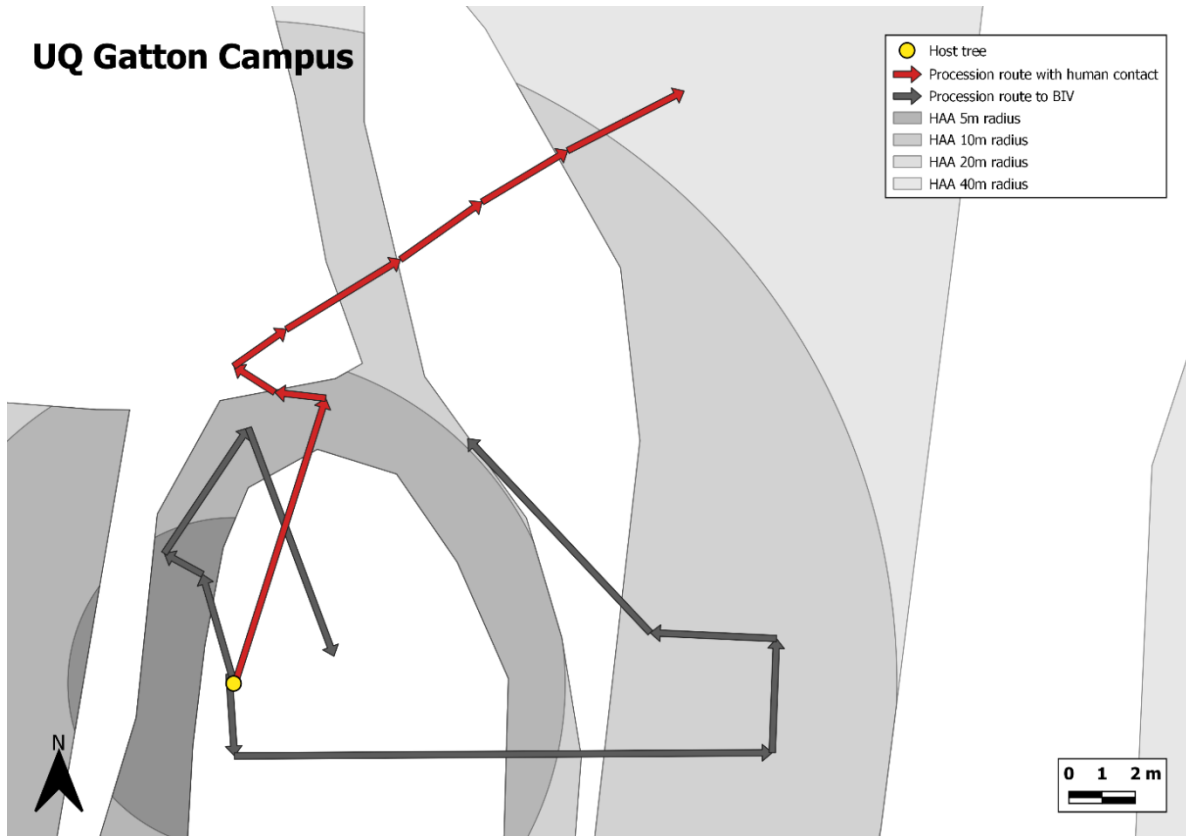

C

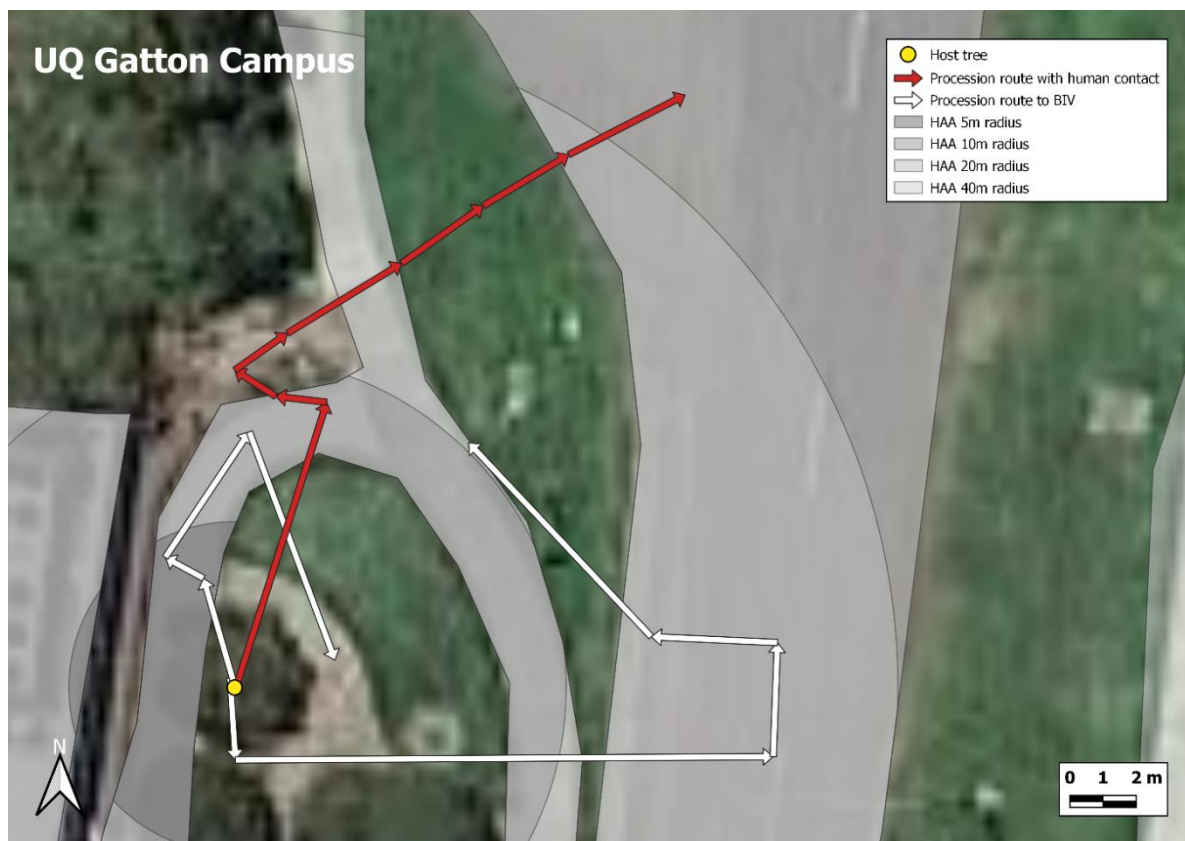

Supplement: Supplementary file 6 — Additional file 6 Images PDF A sub-sample of three Ochrogaster lunifer pre-pupation procession routes from one host tree at the University of Queensland, Gatton campus, Australia. Every procession starts from the host tree (yellow circle) and each arrow represents a change in procession orientation in search for a pupation site (grey/white arrows)/had human contact (red arrows). The tip of the last arrow is the last point. Red arrows represent the procession that was run over by a car on the road (human contact). Grey (A and B)/white (C) arrows represent processions that successfully went into a bivouac. In B and C, various shades of grey circles starting from the host tree is the amount of human attended areas (urban structures) there are for a given radius of various increments (5, 10, 20, 40 m). Additional file 6 A, B and C represents the same three O. lunifer pre-pupation processions with different geographic layers, starting from A being the simplest to C being the most complex with the satellite image. [file 40462_2020_189_MOESM6_ESM.pdf]

A

## Monte Garzon

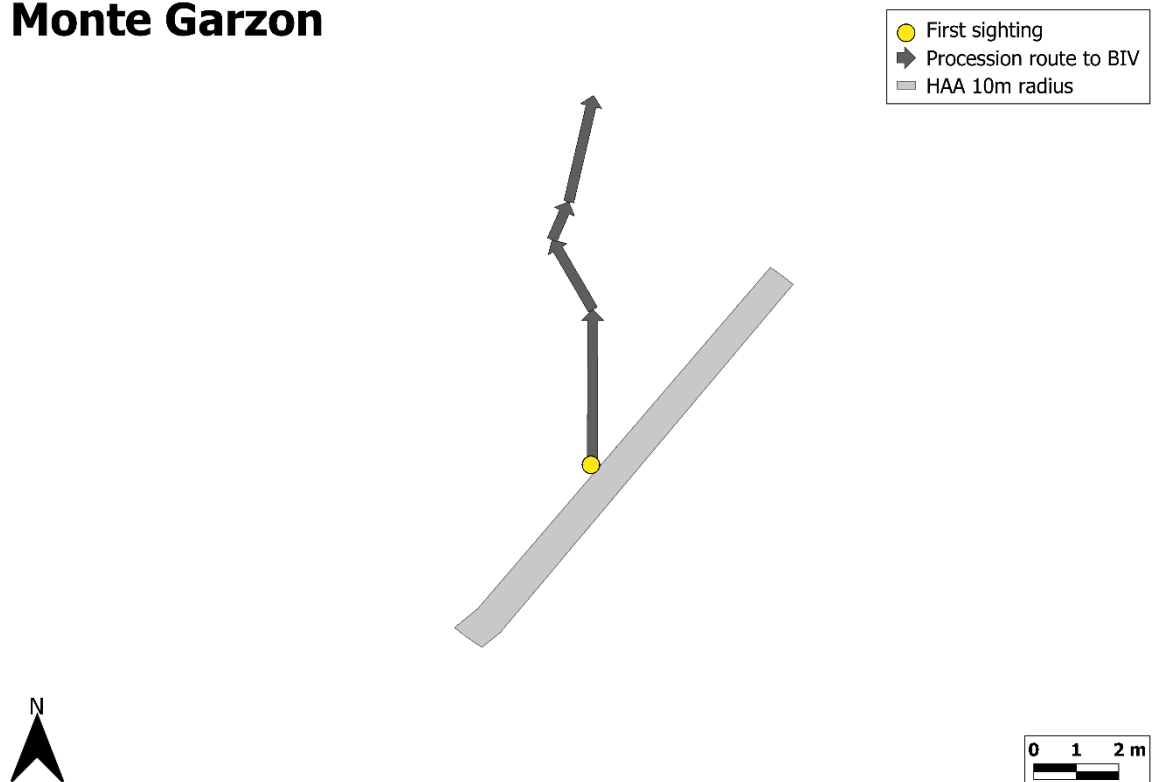

B

## Monte Garzon

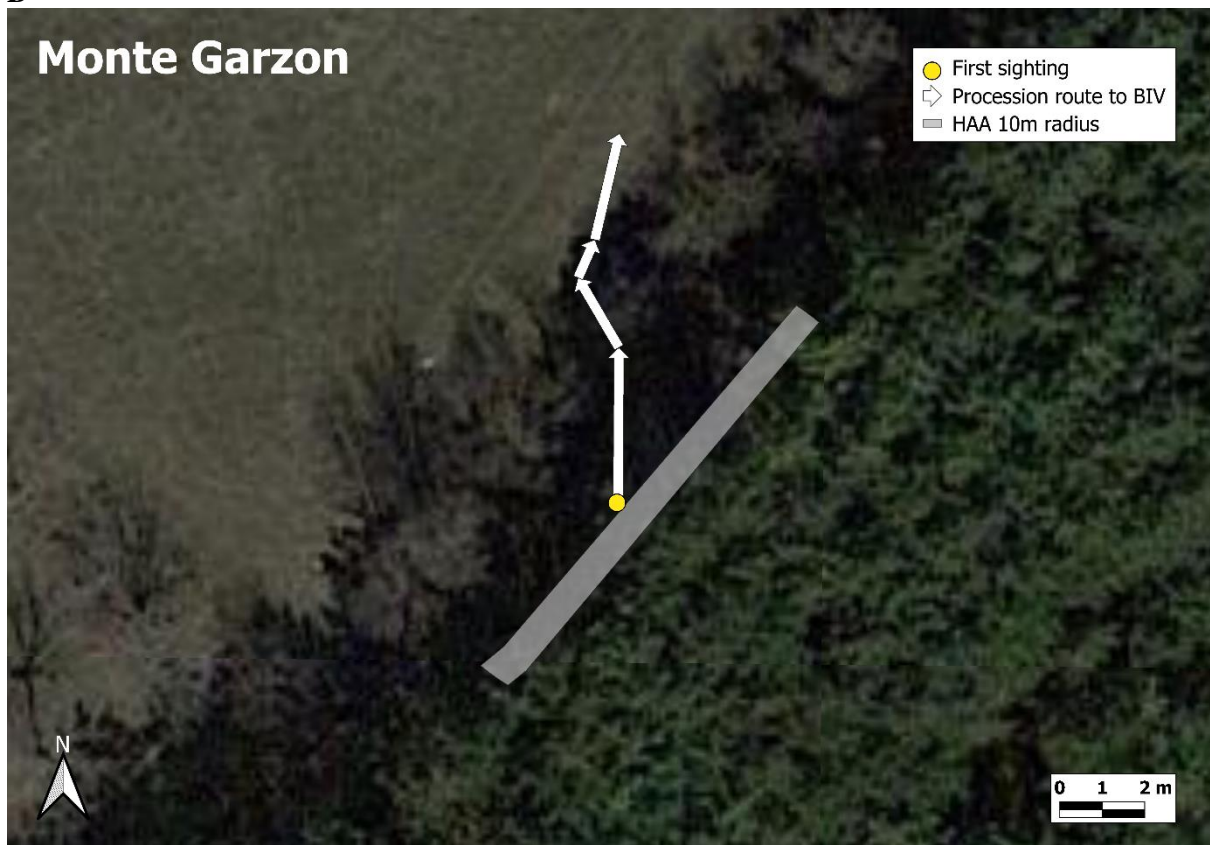

Supplement: Supplementary file 7 — Additional file 7 Images PDF. A Thaumetopoea pityocampa pre-pupation procession route from first sighting along the footpath at Monte Garzon, Veneto Italy. The procession was studied from the first sighting (yellow circle) and every grey (A)/white (B) arrow represents a change in orientation in search for a pupation site. The thick grey line represents the human attended area of a 10 m radius from the first sighting of the procession. Additional file 7 A and B represents the same T. pityocampa pre-pupation procession with different geographic layers, A being the simplest and B being the most complex with the satellite image. [file 40462_2020_189_MOESM7_ESM.pdf]
